# Zinc transporter ZIP10 forms a heteromer with ZIP6 which regulates embryonic development and cell migration

**DOI:** 10.1042/BCJ20160388

**Published:** 2016-08-11

**Authors:** Kathryn M. Taylor, Issa A. Muraina, Dylan Brethour, Gerold Schmitt-Ulms, Thirayost Nimmanon, Silvia Ziliotto, Peter Kille, Christer Hogstrand

**Affiliations:** * Breast Cancer Molecular Pharmacology Unit, School of Pharmacy and Pharmaceutical Sciences, Redwood Building, Cardiff University, King Edward VII^th^ Avenue, Cardiff CF10 3NB, U.K.; † King's College London, Faculty of Life Sciences and Medicine, Diabetes and Nutritional Sciences, Metal Metabolism Group, 150 Stamford St., London SE1 NH9, U.K.; ‡ National Veterinary Research Institute, PMB 01 Vom, Nigeria; § Tanz Centre for Research in Neurodegenerative Diseases, University of Toronto, Toronto, Ontario, Canada M5T 3S8; ║ Department of Laboratory Medicine and Pathobiology, University of Toronto, Toronto, Ontario, Canada M5S 3H2; ¶ Department of Pathology, Phramongkutklao College of Medicine, 315 Ratchawithi Road, Thung Phayathai, Ratchathewi, Bangkok 10400, Thailand; ** School of Biosciences, Cardiff University, Sir Martin Evans Building, Museum Avenue, Cardiff CF10 3AT, U.K.

**Keywords:** cancer, human, mouse, SLC39A10, SLC39A6, zebrafish

## Abstract

There is growing evidence that zinc and its transporters are involved in cell migration during development and in cancer. In the present study, we show that zinc transporter ZIP10 (SLC39A10) stimulates cell motility and proliferation, both in mammalian cells and in the zebrafish embryo. This is associated with inactivation of GSK (glycogen synthase kinase)-3α and -3β and down-regulation of E-cadherin (CDH1). Morpholino-mediated knockdown of *zip10* causes delayed epiboly and deformities of the head, eye, heart and tail. Furthermore, *zip10* deficiency results in overexpression of *cdh1*, *zip6* and *stat3*, the latter gene product driving transcription of both *zip6* and *zip10*. The non-redundant requirement of Zip6 and Zip10 for epithelial to mesenchymal transition (EMT) is consistent with our finding that they exist as a heteromer. We postulate that a subset of ZIPs carrying prion protein (PrP)-like ectodomains, including ZIP6 and ZIP10, are integral to cellular pathways and plasticity programmes, such as EMT.

## Abstract (precis)

Zinc is involved in cell migration during embryo development and in cancer. We show that a zinc transporter consisting of two proteins, ZIP6 and ZIP10, stimulates both cell migration and division in mammalian cells and in the zebrafish embryo.

## INTRODUCTION

As early as 1923 it was reported that tumour tissues of different origin contained aberrant levels of zinc [1]. Thirty-two years later, Tupper et al. [2] published that the uptake of ^65^Zn(II) was higher in mouse mammary tumour tissue compared with non-tumour tissue. The biological explanation for these observations has only been revealed recently as zinc(II) is now known to be an intracellular signalling ion regulating major signalling pathways [3–6]. Several research articles have demonstrated a role of zinc(II) signals in the increased phosphorylation of kinases involved in pathways stimulating cell proliferation and migration [5,7–9]. However, rather than activating kinases directly, it appears that zinc(II) acts primarily through the inhibition of protein phosphatases that dephosphorylate kinases [10–14].

Zinc signals can be initiated by release of zinc from proteins, notably metallothioneins, or by influx into the cytosol through zinc transporters [3,5,15,16]. There is a growing body of literature linking dysregulation of zinc transporters of the SLC39A (Zrt- and Irt-like protein, ZIP) family to various forms of cancer. Elevated expression of ZIP4 is associated with cancers of the liver, pancreas and brain [17–19]. Expression of *ZIP10* mRNA is associated with aggressiveness of renal cell carcinoma [20] whereas a common polymorphic variant of ZIP6 is associated with survival-time in patients with oesophageal squamous-cell carcinoma [21]. Furthermore, expression of the closely related ZIP6 and ZIP10 is high in some breast cancers and contributes to their aggressive behaviour [22–27]. ZIP10 has also been found to be over-expressed in breast cancer cells that metastasis to the lymph nodes [28]. Knockdown of ZIP10 in invasive and metastatic breast cancer cell lines (MDA-MB-231 and MDA-MB-435S) or treatment of the cells with a cell-permeable zinc chelator suppressed cell migration suggesting that ZIP10 stimulated migratory behaviour through its zinc transporting activity [28]. More recently it was shown that ZIP10 is transcriptionally regulated by signal transducer and activator of transcription 3 (STAT3) and STAT5, and suppresses apoptosis in human B-cell lymphoma [6]. Thus, both ZIP6 and ZIP10 are associated with aggressive behaviour in cancerous cells and are regulated by STAT3/5 acting on cognate *cis*-elements in their respective promoters.

Epithelial–mesenchymal transition (EMT) is a central event during cancer metastasis, a process that has been shown to be regulated, in human breast cancer MCF-7 cells, by ZIP6 down-regulation of E-cadherin (CDH1) [23]. EMT is also a normal biological process being involved in cellular events such as gastrulation and tissue regeneration [29]. During EMT programmes, typical cell–cell adhesive interactions are altered such that connections between cells and their local environment become weaker; the cells also acquire a more mesenchymal, spindle-shaped morphology as a result of cytoskeletal rearrangements. EMT is initiated by a number of factors, one of which is STAT3 [30]. Stat3 promotes expression of *zip6* in the zebrafish gastrula organizer, which in turn is essential for the cell autonomous role of Stat3 in EMT of these cells. Zip6 was shown to cause nuclear localization of snail family zinc finger 1 (Snail1), which is a master regulator of EMT [30], leading to repression of *cdh1* expression [4].

In the present study, we show that ZIP10 stimulates EMT and cell migration in human MCF-7 breast cancer cells as well as in the zebrafish embryo in a comparable manner to that previously shown for ZIP6 [4,23,31]. During gastrulation of zebrafish it appears that both Zip6 [4] and Zip10 are necessary for cells to undergo EMT, suggesting that they operate as a unit. In support of this hypothesis we demonstrate that ZIP10 forms a heteromer with ZIP6 explaining their non-redundant requirement for these processes.

## MATERIALS AND METHODS

### Sequence analysis

A multiple sequence alignment was generated with ClustalW incorporating phylogenetically related amino acid sequences of metal transporters, including the 14 human ZIPs, zebrafish Zip10 and iron(II) transport protein 1 (IRT1) from *Arabidopsis thaliana*. Their evolutionary history was inferred by using the maximum likelihood method based on the Le_Gascuel_2008 model [32]. Initial tree(s) for the heuristic search were obtained automatically by applying Neighbor-Join and BioNJ algorithms to a matrix of pairwise distances estimated using a JTT model, and then selecting the topology with superior log likelihood value. The analysis involved 16 amino acid sequences. All positions containing gaps and missing data were eliminated. There were a total of 198 positions in the final dataset. Alignment and evolutionary analyses were conducted in MEGA6 employing 500 bootstrap replicates with the final tree displayed rooted on AtlRT1 [33]. Protein sequences were obtained from NCBI and are notated as ZIP1 (CAG33498.1), ZIP2 (NP_055394.2), ZIP3 (NP_653165.2), ZIP4 (NP_060237.2), ZIP5 (NP_001128667.1), ZIP6 (NP_036451.3), ZIP7 (NP_001070984.1), ZIP8 (NP_071437.3), ZIP9 (NP_060845.2), ZIP10 (NP_001120729.1), ZIP11(NP_001153242.1), ZIP12 (NP_001138667.1), ZIP13 (NP_001121697.1), ZIP14 (NP_001121903.1), DrZip10 (NP_956965.1) and AtIRT1 (AEE84216.1). Potential PEST proteolytic cleavage sites [34] were identified using the epestfind tool within the European Molecular Biology Open Software Suite (EMBOSS) [35].

### Antibodies

ZIP6 and ZIP10 antibodies were generated in-house; anti-rabbit phospho-GSK-3α/β (Ser^21/9^) Antibody #9331 was from Cell Signalling Technology; anti-rabbit E-cadherin (H-108) was from Santa Cruz Biotechnology; mouse anti-V5 antibody was from Invitrogen; rabbit anti-V5 antibody was from Bethyl Laboratories; rabbit anti-paxillin monoclonal antibody Y113 and Cdh1 rabbit polyclonal antibodies to Cdh1 in zebrafish were sourced from Abcam® (ab32084; ab53033) and β-actin in zebrafish was probed with a rabbit monoclonal antibody (Sigma®, A2066). The secondary antibodies used in zebrafish experiments were goat anti-rabbit IgG conjugated to either Alexa Fluor 488 (Abcam®, ab15007) or horseradish peroxidase (GE Healthcare Life Sciences, RPN2108).

### Animals and husbandry

The animal experiments were carried out under a project licence (PPL70/6379) in accordance with the UK Home Office Animals (Scientific Procedures) Act, 1986. Zebrafish embryos were obtained from breeding strains of King's Wild-Type (KWT2) adult fish. Brood stock fish were maintained in a stand-alone tank system containing system water, which was made from reverse osmosis deionized water supplemented with 60 mg/l of sea salt (Tropical Marine®) and 200 μM CaCl_2_ (VWR), giving a conductivity of 250–350 μS. The fish were reared at a temperature of 28.5±0.5°C under a photoperiod regime of 14 h of light and 10 h of darkness. The system water was partially renewed every other day to maintain nitrogenous waste products at minimal levels (ammonia <0.02 mg/l, nitrite <1 mg/l, nitrate <50 mg/l). Fish were fed twice a day with tropical fish flakes (Aquarian). In preparation for breeding, fish were fed either live nauplii brine shrimp larvae (*Artemia salina*) or frozen brine shrimp to improve spawning.

Fertilized eggs were produced either by pair-wise breeding method using a breeding tank containing a male and a female or by colony breeding method placing a box of marbles inside a tank with up to 25 fish. The tanks with breeding fish were inspected the following morning, 0.5–1 h after light was switched on, for embryos that were collected and used for experimentation.

### Generation of Zip10 (Slc39a10) morphants

Antisense morpholino modified oligonucleotides (morpholinos) for zebrafish *zip10* were designed and procured from GENE TOOLS. The nucleotide sequences of the morpholinos used are shown in Table 1. Because of the potential problem with off-target effects produced by some morpholinos [36], both translational blocking and splice blocking morpholinos were designed for *zip10* in addition to a *p53* translational blocking morpholino used for co-injection with *zip10* morpholinos to suppress potential *p53*-mediated apoptosis [37,38]. Zebrafish embryos were micro-injected at the 1–4 cell developmental stage with 1–2 nl of 2–4 ng of each morpholino as previously described [39] and the development of embryos was monitored by inverted light microscope. The embryo morphants (MO) were collected and incubated in ‘fish water’ (deionized water containing 60 mg/l of sea salt; 200 μM CaCl_2_; 0.01% Methylene Blue) at 28.5°C and the stages of development compared with those of un-injected wild-type controls and mismatch morpholino injected controls (i.e. scrambled sequence) (Table 1). In some experiments, the *p53* translational blocking morpholino was co-injected with either of the two types of *zip10* morpholinos at a ratio of 1.5:1.0 and the effect on embryonic development was compared with that resulting from injection of either of the *zip10* morpholinos alone without p53 knockdown.

**Table 1:** Sequences of morpholinos used for gene knockdown experiment Abbreviations: TB, translation blocker; SB, Splice junction blocker.

MO type	Sequence (5′–3′)
*zip10* TB	TGGTATGTGTGTGAACTCTCATCAT
*zip10* SB	ATCACAGCACTGAGACTCACCTCTT
*p53* TB	GCGCCATTGCTTTGCAAGAATTG
Random-control-MASO	scrambled

### Cell line culture

Wild-type MCF-7 breast cancer cells, a gift from AstraZeneca, were cultured in phenol-red-free RPMI 1640 with 5% (v/v) foetal calf serum plus 200 mM L-glutamine, 10 IU/ml penicillin, 10 μg/ml streptomycin and 2.5 μg/ml fungizone at 37°C in a humidified 5% CO_2_ atmosphere. Tissue culture media and constituents were obtained from Life Technologies Europe, plasticware from Nunc. Chinese-hamster ovary (CHO) cells were maintained in minimal essential medium, α-modification (Sigma) with 10% (v/v) foetal calf serum, 4 mM glutamine, 10 IU/ml penicillin, 10 μg/ml streptomycin and 2.5 μg/ml fungizone under 5% CO_2_ at 37°C as previously described [40]. Epithelial mouse NMuMG cells (CRL-1636, A.T.C.C.) were maintained as per the A.T.C.C. distributor's recommendations.

### CRISPR/Cas9 mediated ZIP6 knockout

The CRISPR/Cas9-based ZIP6 knockout (ko) clones (ZIP6^0/0^) were generated in NMuMG cells by introducing single-strand genomic cuts within opposite strands of the first coding exon of the mouse *Zip6* gene using a Cas9 nickase. Cell autonomous non-homologous end-joining then led to frame shifts that generated premature nonsense codons giving rise to non-productive *Zip6* mRNAs subjected to nonsense-mediated decay. The NMuMG ZIP6 ko clone was characterized by western blot analysis and genomic sequencing.

### Affinity capture and quantitative mass spectrometry

Wild-type mouse NMuMG cells or ZIP6^0/0^ cells derived from them by CRISPR/Cas9-based technology were grown to near-confluency, *in vivo* cross-linked in the presence of 1% formaldehyde in PBS and lysed in the presence of Lysis buffer, which consisted of 1% NP-40, 1 mM EGTA, 10% glycerol, 20 mM NaF, 100 mM NaCl, 1× protease inhibitor cocktail (Complete; Roche), 1× phosphatase inhibitor cocktail (PhosSTOP; Roche) in 150 mM Hepes, pH 7.3. The capture of the ZIP6 bait and its co-isolating binders was based on affinity matrices generated by the non-convalent saturation of Protein A agarose with in-house generated rabbit polyclonal anti-ZIP6 antibodies. Following extensive washes with Lysis buffer, binders to the affinity matrix were eluted in 0.2% trifluoroacetic acid and 20% acetonitrile. Subsequently, samples were denatured in 9 M urea, reduced in 5 mM tris-(2-carboxyethyl) phosphine (Sigma–Aldrich), alkylated with 4-vinylpyridine (Sigma–Aldrich) and trypsinized with sequence-grade modified porcine trypsin (Promega). Digests were isobarically labelled with six-plex tandem mass tag (TMT) reagents before in-depth nano-ESI analysis on an Orbitrap Fusion Tribrid mass spectrometer.

Raw mass spectrometry data files were analysed as described previously (PMID: 25490046). Briefly, Mascot (Version 2.4; Matrix Science) and Sequest HT search engines within Proteome Discoverer software (Version 1.4; Thermo Fisher Scientific) were employed to match raw spectra to peptides in the mouse international protein index (IPI) (release 3.87). Search parameters were set to tolerate up to two missed tryptic cleavages. With 4-vinylpyridine used as the alkylating agent, searches assumed all cysteine side chains were pyridylethyl-derivatized. Variable modifications considered were TMT reagent modifications of primary amines, oxidations of methionines, deamidations of asparagine and glutamine, as well as phosphorylations of serines, theonines and tyrosines. For relative quantification, low mass TMT signature ion distributions were analysed by Proteome Discoverer software using an embedded functionality, which also generated the graphs depicting the enrichment ratios of selected proteins. Note that the full interactome dataset will be made available and presented in a separate article.

### Invasion assays

Invasion assays of MCF-7 cells across Matrigel have been described previously [34]. In this case, transiently transfected cells were transferred to invasion chambers 8 h post transfection and harvested 16 h later.

The generation of recombinant expression constructs for *ZIP6* [41], *ZIP7* [40] and *ZIP14* [41,42] with C-terminal V5 tags using vector pcDNA3.1/V5-His-TOPO (Invitrogen) and the LacZ control has already been described. Cells were transfected with the relevant constructs using Lipofectamine 2000 (Life Technologies), according to the manufacturer's instructions. Briefly, 9×10^5^ cells, grown on 60 mm dishes for 24 h, were transfected with 8.8 μg of DNA and 27.5 μl of reagent free of serum and antibiotics. Serum was added to the cells during transfection and 3 mM sodium butyrate was added to cells transfected with constructs encoding ZIP6 and ZIP10 for 14 h prior to harvest.

### Quantitative real-time PCR

Injected zebrafish embryos were observed for epiboly and gastrulation cell movements up to 10 h post fertilization (hpf), the tail bud stage that signifies the end of gastrulation stage. Total RNA was isolated from 10 hpf embryos using TRIzol® (Invitrogen™) according to the manufacturer's protocol. Following first strand cDNA synthesis using the High Capacity Reverse Transcription kit (Applied Biosystems™), gene expression levels were analysed by quantitative real-time PCR (qPCR) on ABI Prism 7700HT Sequence Detection System using a hydrolysis probe assay. The probe and primer sets for genes of interest were designed using Roche Universal Probe Library software (www.universalprobelibrary.com) and the sequences are shown in Table 2. 18s rRNA was used as housekeeping gene as described before [43]. Cycle thresholds (Ct) were obtained for each of the test genes and for the housekeeping gene and the differences between these (ΔCt) calculated. The ΔCt values were transformed into absolute values according to the 2^(−ΔΔCt)^ calculation [44–46]. Primer pairs for each gene were amplified with equal efficiency, as verified using serial dilutions of the respective cDNA. Average expression data per gene were calculated and expressed as log_2_ fold change relative to WT fish.

**Table 2:** Zinc transporter genes and genes of other molecules associated with EMT with their qPCR primer sequences, UPL probe number, product lengths and accession number F, R and P denote forward primer, reverse primer and probe sequences. Tm, melting temperature

Gene	qPCR primer/probe sequences (5′–3′)	Tm (°C)	Probe#	Amplicon size (bp)	Accession no.
*cdh1*	F: tgtcagagttgagcgtgtcc	60	6	93	NM_131820.1
	R: ggaataatccaacctctctttactctt	59			
	P: ttcctctg				
*stat3*	F: gtgtgtattgacaaggagtcaggt	59	46	64	NP_571554
	R: ggatgttgaacttgcgtgaa	59			
	P: gcagccat				
*zip6*	F: gaacgcgcttactttcgagt	59	49	94	NM_001001591.1
	R: acagcagtgccagtgacatc	59			
	P: tggtggcc				
*zip10*	F: gctgttactgctggcatgttt	60	145	73	NM_200671.1
	R: cactgtcaccgtgaagcatt	59			
	P: tgttgcca				
*18s*	F: aaactgtttcccatcaacgag	59	48	67	FJ915075.1
	R: gggacttaatcaacgcaagc	59			
	P: ttcccagt				
*Snai 3*	F: gtgcaagctttgtggaaagg	60	80	88	NM_001077385.1
	R: gcacgtgaatggtttctcac	59			
	P: tctccagg				

### *In situ* hybridization

Assay of mRNA expression for *cdh1* was performed on whole mount embryos at 10 hpf by *in situ* hybridization (ISH) as described before [47].

### Immunocytochemistry and immunohistochemistry

For immunofluorescence, 2.2×10^5^ cells were seeded on 0.17 mm thick coverslips for 24 h prior to transfection. Cells were fixed with 4% formaldehyde for 15 min, permeabilized or not with 0.4% saponin in 1% BSA in 1× PBS for 15 min, blocked with 10% normal goat serum, incubated with primary antibodies at room temperature, Alexa 594 followed by Alexa 488 anti-mouse or rabbit (1/2000, Molecular Probes) for 30 min, and assembled on to slides using Vectorshield with DAPI (Vector Laboratories). Coverslips were viewed on a Leica RPE automatic microscope using a 63× oil immersion lens. The fluorescent superimposed images were acquired using a multiple bandpass filter set appropriate for DAPI and fluorescein as well as bright field for differential interference contrast. All images were processed with one level of deconvolution using Openlab software (Improvision) to aid clarity. The brightfield images were obtained using the differential interference contrast capability.

For zebrafish immunofluorescence histochemistry, embryos were collected at 10 hpf (tail-bud stage), dechorionated and fixed overnight in 4% paraformaldehyde (PFA) at 4°C. Fixed embryos were rinsed three times at 5 min intervals using ice-cold 1× PBS and subsequently permeabilized by emersion in 100% methanol at −20°C for 10 min before rinsing in 1× PBS for a further 5 min. The embryos were then blocked in 1% BSA diluted in PBS with 0.1% (v/v) Tween 20 (PBST) for 30 min and incubated overnight at 4°C in diluted primary antibody with 1% BSA. The antibody solution was removed and the embryos washed three times in 1× PBS at 5 min intervals. Thereafter, the embryos were incubated in the dark for 1 h at room temperature in diluted secondary antibody conjugated with Alexa Fluor 488 with 1% BSA. The primary antibody to *cdh1* (Anti-Cdh1 rabbit polyclonal; Abcam®, ab53033) was used at 1:100 whereas the secondary antibody (goat anti-rabbit conjugated to Alexa Fluor 488; Abcam®, ab15007) was used at 1:300. Expression and cellular location of Cdh1 protein in stained embryos was observed under laser scanning confocal microscope (Leica, DMIRE2), 2-photon laser scanning confocal microscope (Nikon; Eclipse Ni-EFN Upright/A1R Si MP Confocal) and epi-fluorescence microscope (Nikon eclipse 400).

### SDS/PAGE and western analysis

Cells were harvested, washed with PBS, lysed for 1 h at 4°C with 5.5 mM EDTA/0.6% Nonidet P40/10% mammalian protease inhibitor cocktail (Sigma–Aldrich) in Krebs–Ringer HEPES buffer [40] and centrifuged at 10000 ***g*** for 15 min at 4°C. Protein was measured using the DC assay kit (Bio-Rad Laboratories). Transfected CHO cells were harvested between 16 and 24 h after transfection as described previously [40]. Primary antibodies used were diluted ZIP6-SC, ZIP6-M and ZIP6-Y 1/1000, V5 1/2000, β-actin 1/10,000. Quantification of western blot results was performed by normalization of three separate experiments to β-actin values [40].

For analysis of zebrafish proteins by western blot, embryos were collected at 10 hpf (tail-bud stage), washed in PBS, dechorionated and deyolked in a solution containing Ringer solution with the addition of 10% EDTA (10 mM) and PMSF protease inhibitor at 0.3 mM final concentration. Thereafter de-yolked embryos were collected in Eppendorf tube, rinsed twice in cold Ringer's solution and homogenized at 4°C in lysis buffer containing 1× PBS and PMSF protease inhibitor cocktail (Sigma) in the ratios of 90:10 μl. Tissue lysates were then centrifuged at 1000 rpm at 4°C for 5 min and the supernatant was transferred to a fresh tube. Total protein was quantified by Pierce assay kit-rapid (51254-1KT, Sigma) and subjected to SDS/PAGE western blot using standard procedures. Primary anti-Cdh1 antibody (rabbit polyclonal, Abcam®, ab53033) was used at 1:1000 and visualized using ECL with goat anti-rabbit IgG antibody conjugated to horseradish peroxidase used at 1:7500 (GE Healthcare Life Sciences, RPN2108). β-Actin (rabbit monoclonal; Sigma®, A2066) was also at 1:1000 with a secondary antibody (goat anti-rabbit horseradish peroxidase, GE Healthcare Life Sciences, RPN2108) at 1:2000. Quantification of immunoblot results was performed by normalization of three separate experiments to β-actin values.

### FACS analysis and zinc uptake assay

For cell cycle analysis, adherent cells, harvested carefully by pipette, or non-adherent cells were collected and the analysis was performed using CycleTEST PLUS DNA reagent kit (Becton Dickinson) and following manufacturer's instructions.

For zinc uptake assay, cells were loaded with 5 μM of the zinc indicator, Newport Green diacetate DCF (Invitrogen), treated with 10, 25 or 50 μM zinc plus 10 μM zinc ionophore sodium pyrithione (Sigma–Aldrich) at 37°C for 20 min before reading on a Becton–Dickinson FACS III flow cytometer.

### Proximity ligation assay

MCF-7 cells on an 8-well chamber slide (Lab-Tek, Fisher) were fixed with 3.7% formaldehyde in PBS for 15 min followed by proximity ligation assay (PLA) using Duolink red kit (Sigma–Aldrich) as described previously [5]. Antibodies against ZIP6 (ZIP6-Y, mouse monoclonal, in-house) and ZIP10 (ZIP10-R, rabbit polyclonal, in-house) were applied at 1:100 and 1:100 dilutions, respectively. The slide was mounted with Vectashield with DAPI, and viewed on a Leica RPE microscope with a 63× oil immersion lens. Images were acquired with a multiple band-pass filter set appropriate for DAPI and Texas Red and presented as maximal projections of 25 stacks taken 0.3 μm apart. Dots per cell were determined with ImageTool software (Olink) using at least 12 cells from at least three experiments and presented as average values±S.E.M.

### Statistical analysis

Statistical analysis of cell data was performed using ANOVA with Post-Hoc Dunnett and Tamhane tests. Significance was assumed with *P*<0.05. Values are expressed as means of at least three experiments (*n* ≥ 3) with error bars showing the S.E.M. For zebrafish analysis, data were analysed using one way ANOVA or Student's unpaired *t* tests with significance at *P*<0.05. Values are presented as the mean±S.E.M. (*N*=12).

## RESULTS

### ZIP6 and ZIP10 are closely related paralogues

**Figure 1:**
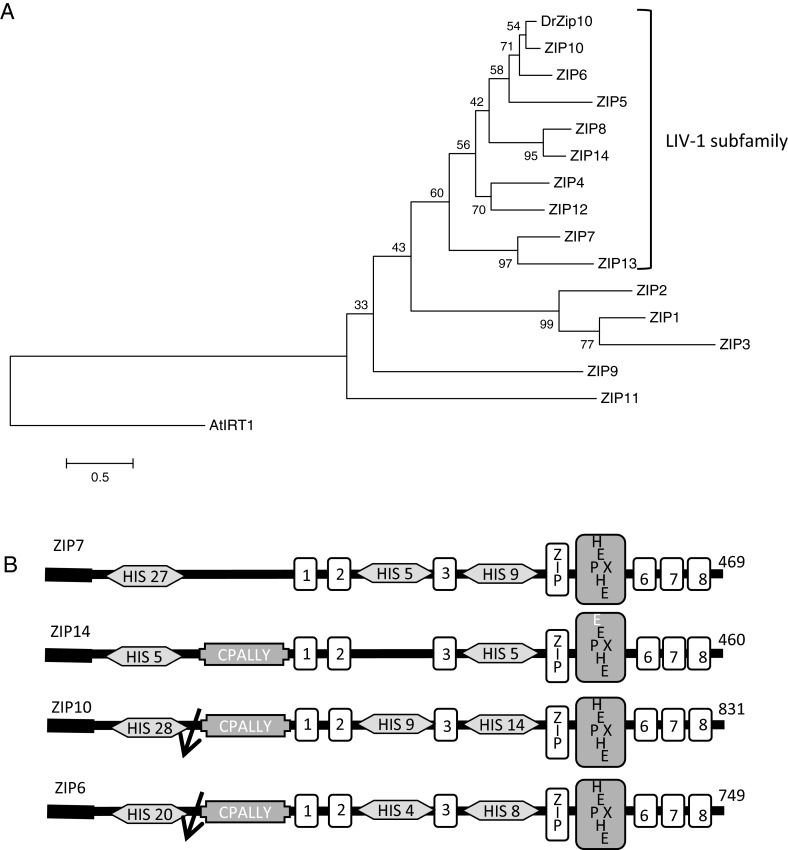
Structural relationships of the human ZIP (SCL39A) family of zinc transporters (**A**) Molecular phylogenetic analysis of human ZIP (SLC39A) family of zinc transporters and zebrafish Zip10 (DrZip10) as generated by the maximum likelihood method. The tree with the highest log likelihood (−5949.0421) is shown. The percentage of trees in which the associated taxa clustered together is shown next to the branches. Sequences belonging to the LIV-1 sub-family of ZIP proteins are denoted. (**B**) Depiction of motifs in selected ZIP proteins from the LIV-1 sub-family. Transmembrane domains are illustrated by rectangular boxes; His: histidine cluster; CPALLY: conserved motif only present in PM-located LIV-1 sub-family members; arrow: proteolytic cleavage site at putative PEST motif; ZIP: ZIP family amino acid signature motif in transmembrane domain 4; HEXPHE: PDF metalloprotease motif in transmembrane domain 5; numerals 1, 2, 3, 6, 7 and 8 in rectangles denote the other transmembrane domains.

Phylogenetic analysis of the amino acid sequences of proteins within the SLC39A (ZIP) family reveals that ZIP6 and ZIP10 are very closely related members of the LIV-1 sub-family (Figure 1A). Furthermore, zebrafish Zip10 is more similar to human ZIP10 than to any other human protein and these two proteins are considered orthologous (Figure 1A) [48]. In addition to the motifs in common to the whole mammalian ZIP protein family, ZIP6 and ZIP10 both have long histidine-rich (20–28 His) N-terminal domains preceding the first transmembrane domain (Figure 1B). Although ZIP7 also has multiple histidines in its N-terminal domain, this region in ZIP7 is considerably shorter than ZIP6 and ZIP10. Another feature of ZIP6 and ZIP10 is the presence of a potential PEST cleavage site before the CPALLY motif, which is found in all LIV-1 sub-family of ZIP proteins except ZIP7 and ZIP13. The potential PEST motif in ZIP10 is located between amino acid residues 206 and 219 (RGEPSNEPSTETNK). Although it is not known if this is an actual site for proteolytic cleavage in ZIP10, we have previously reported experimental evidence that ZIP6 is cleaved at the corresponding predicted PEST site (residues 210–223) in the endoplasmic reticulum (ER) before locating to the plasma membrane (PM) and mediating zinc influx [23].

### ZIP10 mediated zinc influx causes loss of adhesion and stimulates cell proliferation

**Figure 2:**
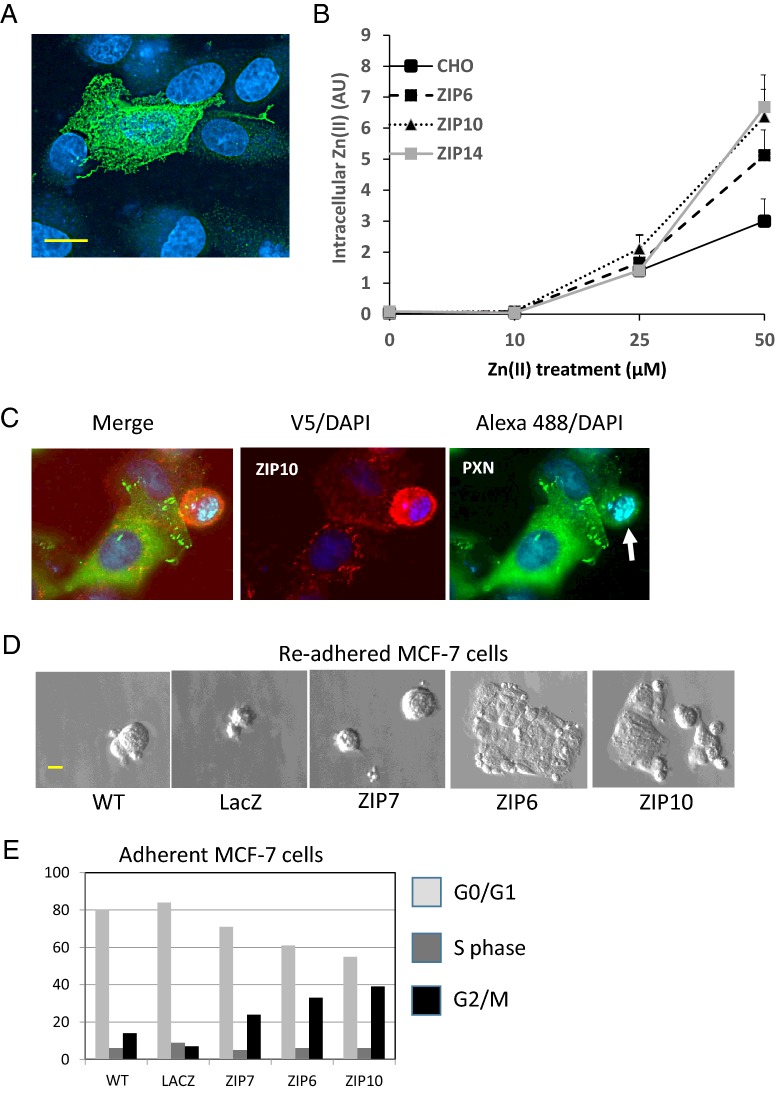
ZIP10 has PM localization, mediates zinc influx and stimulates cell proliferation (**A**) Immunofluorescence of unpermeabilized MCF-7 cells transfected with a human ZIP10 construct and probed with an anti-ZIP10 antibody. Scale bar, 10 μM. (**B**) Uptake of zinc(II) in CHO cells over 20 min at different concentrations of total zinc(II) added to the culture medium (in absence of FBS). The values show a mean of three replicate assays and the error bars represent S.E.M. (**C**) Paxillin focal adhesions are greatly reduced in MCF-7 cells overexpressing exogenous ZIP10 which round up as a consequence. MCF-7 cells were transfected with ZIP10 (V5 tag) and probed for V5-tagged ZIP10 (red) and PXN (green); chromatin was stained with DAPI (blue). PXN focal adhesions are evident in adherent ZIP10 negative or positive cells. Overexpression of ZIP10 causes cells to round up (white arrow) and loose adhesion. These cells have limited or no PXN focal adhesions (white arrow). (**D**) Attachment potential of collected detached and re-seeded MCF-7 cells from cultures of either WT cells, or MCF-7 cells transfected with LacZ (transfection control) or human ZIP7, ZIP6 or ZIP10. Scale bar, 10 μM. (**E**) Cell cycle stage analysis by FACS of either wild-type MCF-7 cells (WT) or MCF-7 cells transfected with LacZ (transfection control) or human ZIP7, ZIP6 or ZIP10. Values are means from three experiments.

Exogenous expression of ZIP10 in cells consistently resulted in a low percentage of cells expressing the protein. Using an antibody directed to the N-terminus, immunofluorescence of ZIP10 in MCF-7 breast cancer cells overexpressing the protein demonstrated PM staining (Figure 2A). Influx of zinc in CHO cells transfected with ZIP6, ZIP10 or ZIP14, was analysed by FACS using the cell-permeant fluorescent zinc probe Newport Green™ (*K*_d_=1 μM). Cells overexpressing either of these transporters showed increased accumulation of chelatable zinc with increasing concentrations of zinc added to the medium, compared with the untransfected CHO cells, over the 20 min transport assay (Figure 2B). Zebrafish Zip10 has previously been shown to be a zinc importer using the Xenopus oocyte expression system [43] and these experiments confirm that human ZIP10 performs the same transporter function.

Exogenous expression of ZIP6 or ZIP10 in MCF-7 cells resulted in a large proportion of the cells rounding up and floating to the surface. This change in morphology was accompanied by loss of paxillin (PXN) focal adhesions, reducing binding to the surface (Figure 2C; Supplementary Figure S1). When these floating cells were collected and re-seeded on coverslips they re-attached and continued to grow (Figure 2D). In contrast, control untransfected or LacZ transfection control cells that detached from the substrate did not easily re-adhere and neither did cells transfected with ZIP7. Intriguingly, in contrast with the ZIP6/ZIP10 which migrate to the PM ZIP7 is only observed located in the ER and never observed in the PM [5]. These results indicate that the PM located zinc importers, ZIP6 and ZIP10, are stimulating the cells to round-up and detach and that this population of floating cells is still viable. Furthermore, FACS analysis of MCF-7 cell cycle transfected with the same constructs showed dramatic effects of ZIP6 and ZIP10 on cell proliferation, increasing the number of cells in G_2_/M phase from 14 to 40% (Figure 2E).

### ZIP10 promotes epithelial–mesenchymal transition in MCF-7 cells

**Figure 3:**
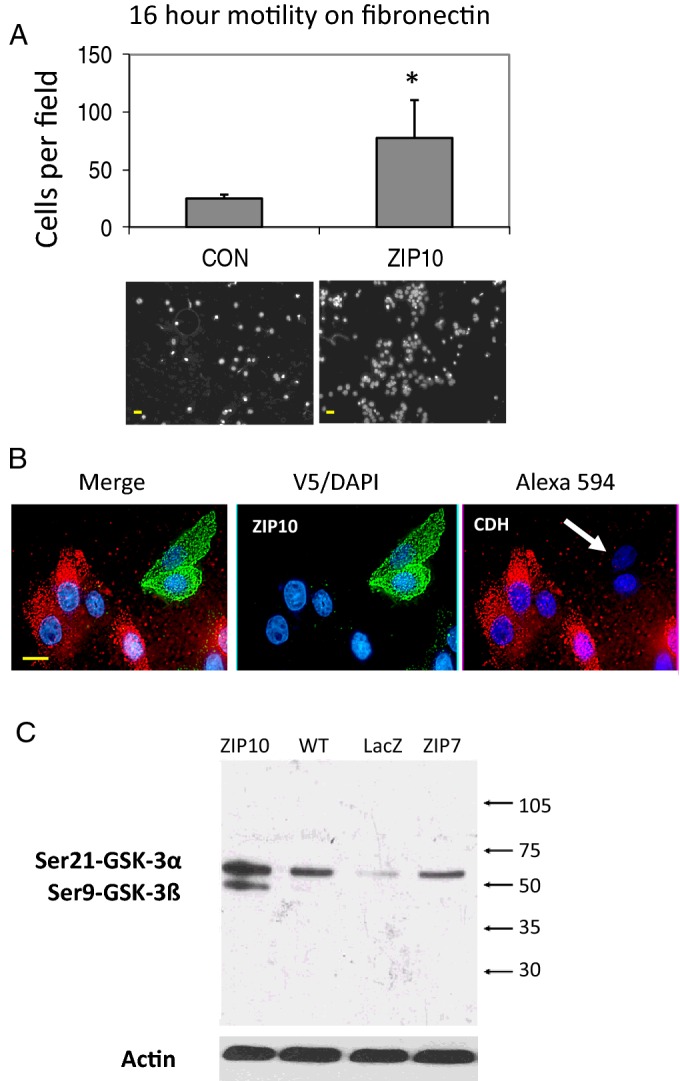
ZIP10 promotes motility of MCF-7 cells through down-regulation of CDH1 and inactivation of GSK-3 (**A**) MCF-7 cells transfected with ZIP6 or ZIP10 for 8 h migrated across Matrigel™ on a Transwell plate over the next 16 h more than controls. Images represent migrated DAPI-stained cells. In each of three experiments, 12 fields of view were assessed and data averaged. Values are presented as means±S.E.M. (*N*=3). The asterisk indicates statistical difference from control at *P*<0.05. Scale bar, 10 μM. (**B**) Loss of CDH1 (red) only in recombinant ZIP10-V5 positive cells probed with anti-V5 antibody (green). Scale bar, 10 μM. (**C**) Representative immunoblot of Ser^21^-GSK-3α and Ser^9^-GSK-3β in wild-type MCF-7 cells (WT) and in MCF-7 cells transfected with LacZ (transfection control), or human ZIP10 or ZIP7. Immunoblot for β-actin was used as loading control.

To investigate whether the rounding up and detachment of cells overexpressing ZIP10 may be due to initiation of EMT we first quantified the migratory potential of MCF-7 cells transfected with ZIP10 over fibronectin substrate and observed that this tripled over 16 h compared with mock-transfected controls (Figure 3A). Previous studies had shown that during EMT expression of E-cadherin (CDH1) was down-regulated [49]. To confirm whether the down-regulation of CDH1 was involved in causing the observed increase in cell migration in response to over-expression of ZIP10, we performed immunofluorescence analysis of V5-tagged ZIP10 and native CDH1 in MCF-7 cells transfected with a ZIP10-V5 construct. Cells that stained positively for ZIP10-V5 showed little or no staining for CDH1, in contrast with ZIP10-V5 negative cells, which abundantly expressed CDH1 (Figure 3B). During EMT GSK-3α and GSK-3β are inactivated through phosphorylation at Ser^21^ and Ser^9^ respectively. ZIP10 transfected cells showed increased abundance of serine phosphorylated GSK-3α (Ser^21^) and of GSK-3β (Ser^9^, Figure 3C). Interestingly, transfection of cells with the ER located ZIP7 had no effect on GSK-3 phosphorylation.

### Zip10 regulates embryonic development of zebrafish

Since all evidence suggested that ZIP10 induced EMT in the human MCF-7 breast cancer cell line, it was of interest to know if this zinc transporter has a similar function in living organisms. Earlier work in zebrafish has demonstrated that Zip6, the closest paralogue of Zip10, regulates EMT in the gastrula organizer [4]. Thus, we investigated the effect of antisense morpholino knockdown of *zip10* mRNA on zebrafish development.

**Figure 4:**
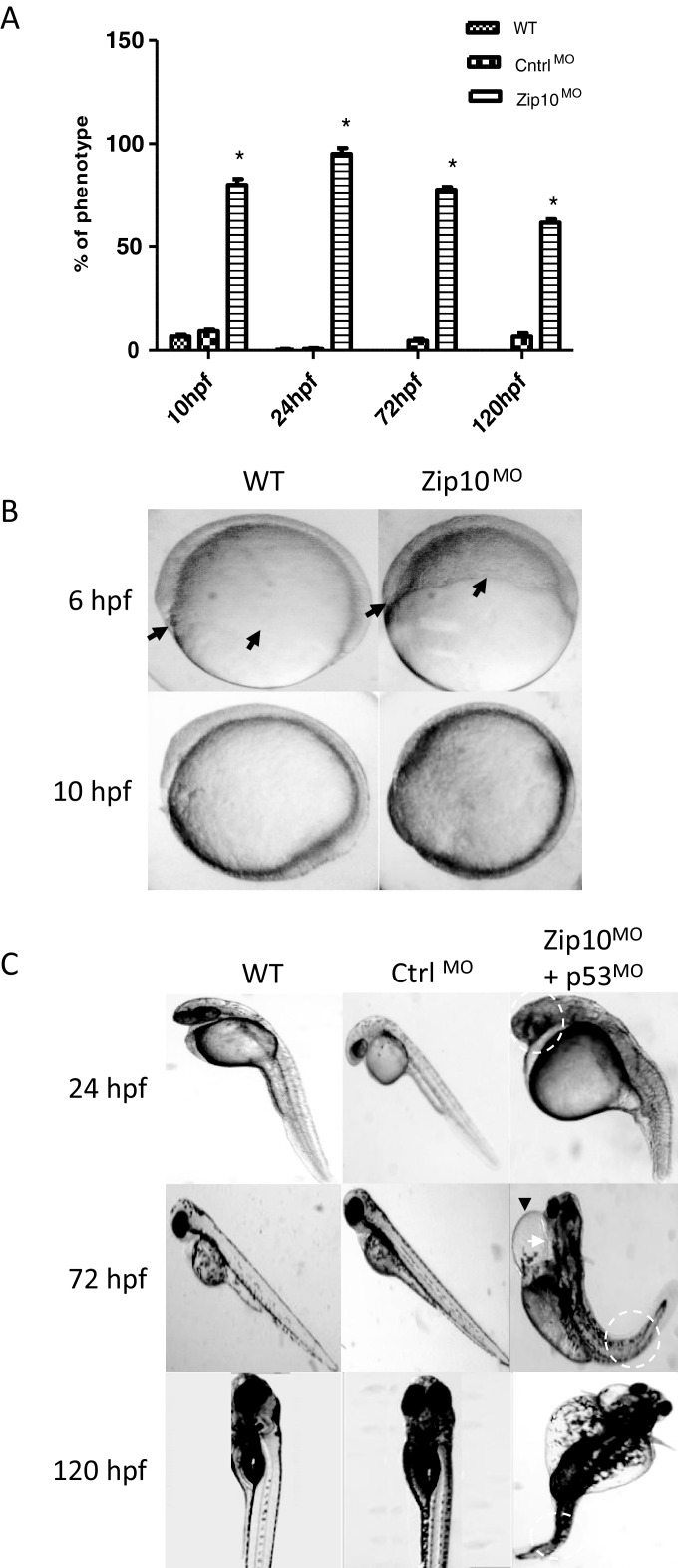
Phenotype of Zip10 knockdown zebrafish embryo Zebrafish embryos were co-injected with 2 ng of translation blocking morpholino-modified oligonucleotides against *zip10* and *p53* (to block off-target effects causing apoptosis; Zip10^MO^) or with 2 ng of a scrambled control morpholino (Ctrl^MO^) and compared with uninjected wild-type (WT) embryos. (**A**) Percentage of embryos displaying morbidities (% phenotypes) at 10, 24, 72 and 120 hpf. (**B**) Epiboly cell migration in wild-type embryos and in embryos injected with a translation blocking morpholino against *zip10* (Zip10^MO^) or with a scrambled control morpholino (Cntrl^MO^). The extension of epipoly migration was delayed in Zip10^MO^ embryos compared with time-matched controls (arrows) at 6 and 10 hpf with resulting delay in tail bud formation in morphants at 10 hpf compared with wild type. Morphant was yet to complete epiboly (85% complete) at 10 hpf when tail bud has already formed in wild type. (**C**) Common phenotypes of Zip10^MO^ embryos at later stages of development (24–120 hpf). Note the Zip10^MO^ embryo at 24 hpf with shorter anterior-posterior axis with abnormal head and eye formation (highlighted with white broken circle). Also note the oedema of the pericardial sac (black arrow-head) and the miss-formed heart (heart-string) feature (white arrow) in 72 hpf Zip10^MO^ embryos. Severe oedema of pericardium and yolk sac was observed in many embryos at 120 hpf. Note also the curled or twisted tail at 72 and 120 hpf (white broken circles). The experiment was carried out five times with 20–50 embryos each time (*N*=5). Results were also replicated using a splicing blocking morpholino against *zip10* (results not shown).

*Zip10* morphants (Zip10^MO^) showed dose-dependent developmental morbidity and few embryos injected with 10 ng of either *zip10* translation-blocking or splice-blocking morpholino developed beyond the early stages of epiboly and, hence, did not gastrulate. At a concentration of 2 ng, embryos were viable but showed distinct phenotype (Figure 4A; Supplementary Figure S2). Similar effects were observed for the *zip10* translation-blocking morpholino or splice-blocking morpholino, whether injected alone (Figures 4A and 4B; Supplementary Figure S2) or co-injected with *p53* morpholino to supress potential off-target induced apoptosis (Figure 4C). Typical developmental abnormalities observed in Zip10^MO^ embryos injected with 2 ng of the translation-blocking morpholino included delayed epiboly and EMT movement (Figure 2B), abnormally small head, small eyes with delayed pigmentation, general under-development with shorter anterior-posterior axis (i.e. runted embryo), coiled or twisted tail, oedema of the pericardial sac with abnormal string-heart, and wide-spread oedema of the embryo post hatching (Figure 4C; Supplementary Figure S2). Some of the Zip10^MO^ were missing the tail altogether. Such effects were uncommon in either un-injected embryos or those injected with a scrambled morpholino sequence (Figure 4A).

**Figure 5:**
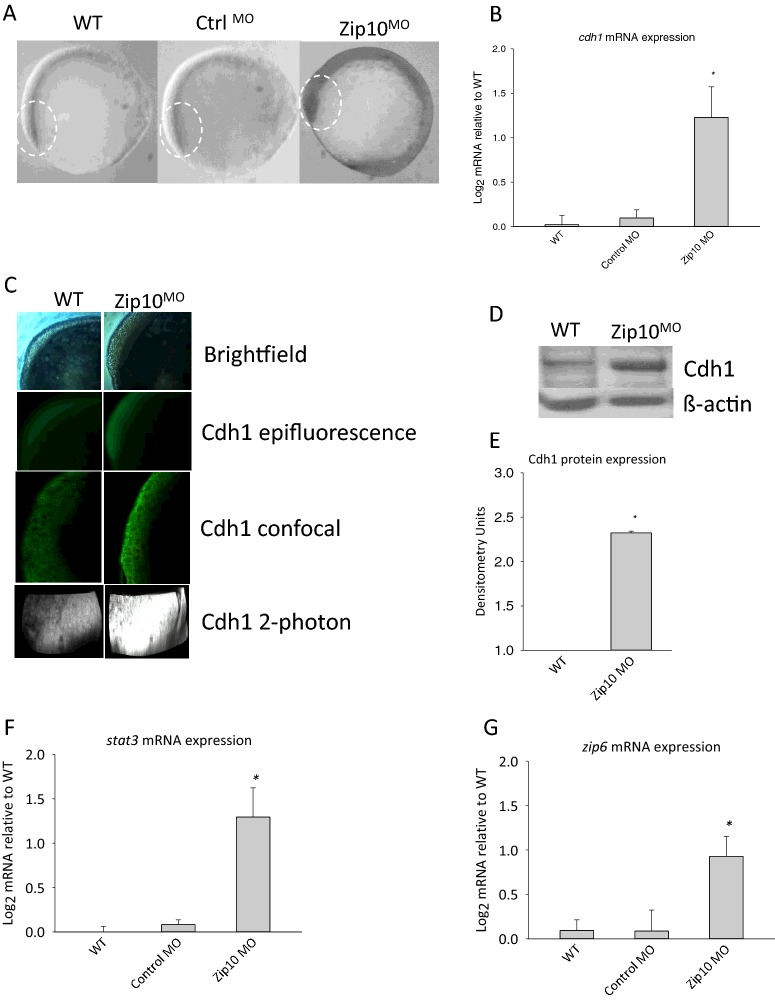
Effect of Zip10 knockdown on expression of genes involved in EMT during gastrulation of the zebrafish embryo 10 hpf (**A**) ISH of E-cadherin (*cdh1*) mRNA in zebrafish wild-type embryos (WT), control embryos injected with a scrambled morpholino (Ctrl^MO^), and in embryos injected at 1–4 cell stage with a translation blocking morpholino against *zip10* (Zip10^MO^) at 10 hpf. Ten embryos from each group were assessed and the experiment was repeated with the same results. (**B**) Expression of *cdh1* mRNA in WT, Cnrtl^MO^ and Zip10^MO^ zebrafish embryos as measured by qPCR. Data are presented as the mean±S.E.M. (*N*=6 and the experiment was repeated with the same results). (**C**) Expression of Cdh1 protein during epipoly in WT and Zip10^MO^ embryos as assessed by immunohistochemistry (*N*=3) or (**D**) by immunoblots, which were (**E**) quantified by band intensity (mean±S.E.M., *N*=3). qPCR analysis of mRNA for the gastrulation-inducing gene products of (**F**) *stat3* and (**G**) *zip6*. Data are expressed as log_2_ fold change and error bars represent mean±S.E.M. (*N*=6 and the experiment was repeated with the same results). Data were statistically evaluated by ANOVA and significant differences (*P*<0.05) relative to the WT indicated by an asterisk.

The delayed epiboly movement of cells over the yolk and/or EMT migration could be observed at 6 and 10 hpf in embryos injected with *zip10* morpholino (Figure 4B). At 10 hpf Zip10^MO^ showed increased expression of *cdh1* mRNA as analysed by ISH (Figure 5A) and qPCR (approximately 2.4-fold increase; Figure 5B). This translated into increased Cdh1 protein expression as indicated by immunohistochemistry techniques (Figure 5C) and confirmed by western blot (Figures 5D and 5E). Interestingly, morpholino knockdown of *zip10* also resulted in increased abundance of *stat3* and *zip6* mRNA in embryos (Figures 5F and 5G).

### ZIP10 forms a heteromeric complex with ZIP6

**Figure 6:**
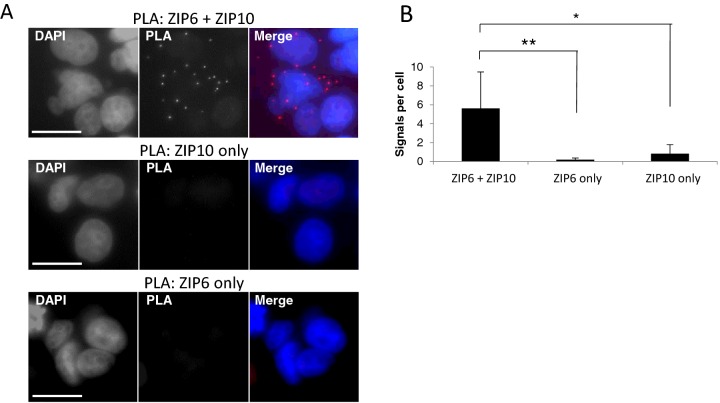
Proximity ligand assay of ZIP6 and ZIP10 in MCF-7 cells (**A**) Proximity ligation assay using ZIP6 and ZIP10 antibodies in MCF-7 cells produces multiple red fluorescent dots indicative of binding, with presence of few dots in ZIP6 only and ZIP10 only controls. (**B**) Quantification of numbers of ZIP6:ZIP10 interaction events per cell showing multi-fold greater numbers of dots when antibodies to both proteins are applied compared to controls where ZIP6 or ZIP10 antibodies are applied separately. Results are demonstrated as mean ±S.D. *P<0.05, **P<0.01. Scale bar, 10 μm.

Results from knockdown experiments in zebrafish embryos suggest collectively that Zip10 is required for EMT and cell motility in the early zebrafish embryo. The same function has been attributed to Zip6 [4] and it, thus, appears that both these proteins are required for induction of EMT during epiboly in zebrafish. Furthermore, we have shown before that ZIP6 stimulates migratory behaviour and EMT also in human cells [23,50]. Therefore, we asked if ZIP6 and ZIP10 might operate as a heteromeric complex and investigated this first through the PLA, which utilizes ‘rolling circle amplification’ of complementary DNA oligonucleotides linked to two separate secondary antibodies raised against primary antibodies from different species [51]. With primary antibodies raised in the corresponding two species against two potentially interacting proteins the two complementary oligonucleotides will hybridize forming a circular DNA if the two proteins reside <40 nm apart. The proximity of the two proteins is visualized by biotin labelling as a dot. Application of either ZIP6-Y or ZIP10-R antibody alone to fixed MCF-7 cells generated less than two dots per cell (Figure 6). In contrast, when both antibodies were applied together the PLA showed on average six dots per cell, indicative of ZIP6:ZIP10 heteromers being present (Figure 6).

**Figure 7:**
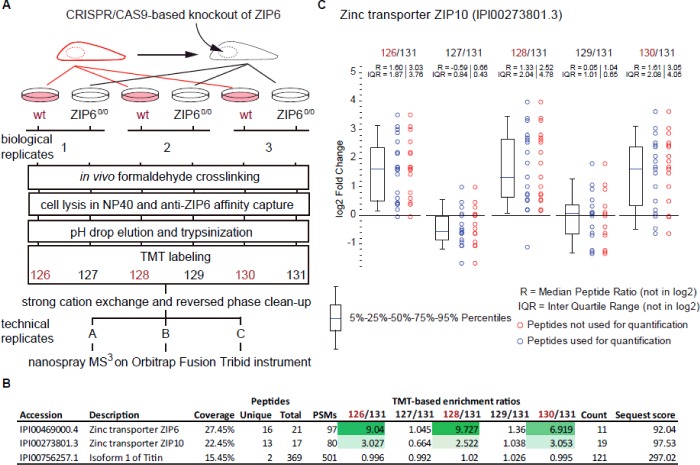
Co-isolation of ZIP6 and ZIP10 by affinity capture followed by quantitative mass spectrometry (**A**) Workflow of quantitative mass spectrometry analysis. Wild-type (wt) mouse NMuMG cells or ZIP6^0/0^ cells were grown to near confluency and *in vivo* cross-linked in the presence of 1% formaldehyde (in PBS) prior to harvest and lysis. The affinity capture was based on identical affinity chromatography matrices generated by the non-covalent capture of an anti-ZIP6 antibody on Protein A agarose. Following extensive washes, ZIP6 and its co-isolating binding partners were eluted by pH drop before samples were denatured in urea, reduced, alkylated and trypsinized. To minimize intra-assay variance and enable the relative quantification of proteins, digests were isobarically labelled with six-plex TMT reagents before in-depth nano-ESI analysis on an Orbitrap Fusion Tribrid mass spectrometer. (**B**) ZIP6 interacts with ZIP10 but not with Titin. Presented are benchmarks of enrichment for a subset of proteins identified in the ZIP6 interactome dataset. (**C**) Chart depicting distributions of TMT signature ion ratios within peptide-to-spectrum matches (PSMs) that supported the identification of ZIP10. Note the selective co-enrichment of ZIP10 only in co-immunoprecipitations from wild-type NMuMG cells.

**Figure 8:**
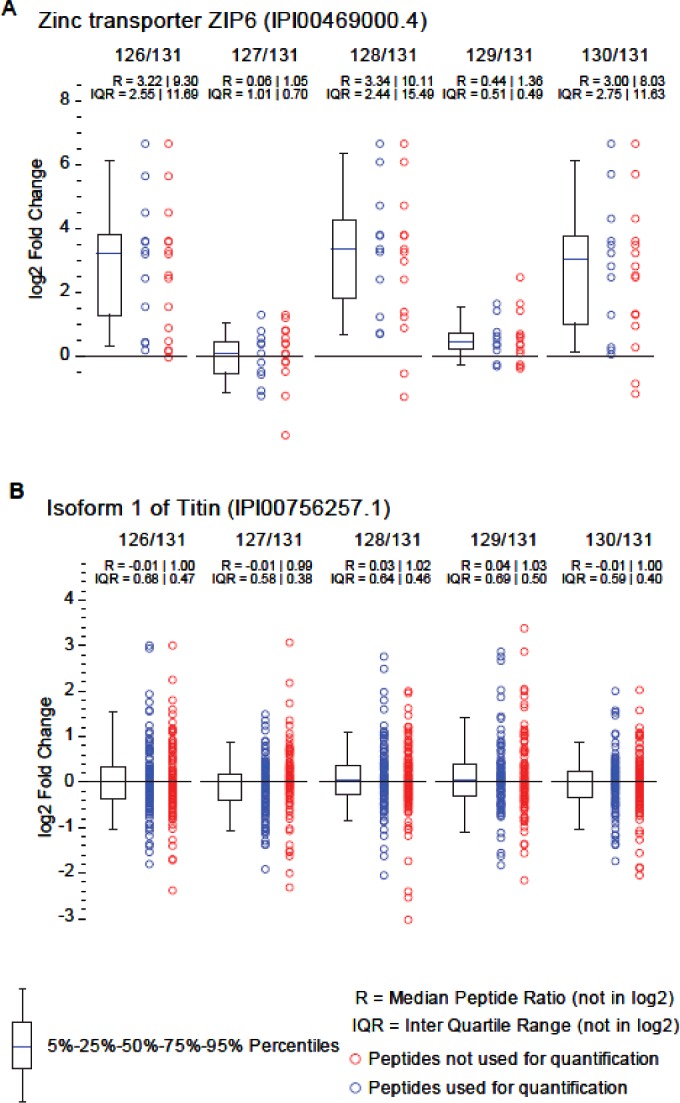
Evidence for selective isolation of the ZIP6 bait protein and non-specific isolation of Titin TMT signature ion ratios derived from peptide-to-spectrum matches (PSMs) that supported the identifications of (**A**) ZIP6 and (**B**) Titin. Note the expected affinity capture of ZIP6 only in immunoprecipitations from wild-type NMuMG cells, and the non-specific co-isolation of Titin in all affinity capture reactions. See Figure 7 for further details.

In search of further evidence for the formation of a ZIP6:ZIP10 complex and in order to establish the presence of this complex in other cell types we generated ZIP6 knockout (ZIP6^0/0^) mouse NMuMG mammary gland cells using the CRISPR/Cas9 system (Figure 7A). Proteins in wild-type (wt) or ZIP6^0/0^ cells were *in vivo* cross-linked, lysed, and subjected to affinity capture using a ZIP6 antibody non-covalently linked to Protein A agarose (Figure 7). ZIP6 and its interacting partners were eluted, trypsinized and peptides quantified in three technical replicates by TMT analysis using six-plex isobaric mass tags to separate the six biological samples. Consistent with expectations, the ZIP6 bait protein was reproducibly observed as the most highly enriched protein in the three biological replicates and this observation was selective for co-immunoprecipitations from wild-type NMuMG cells (Figures 7B and 7C). The identification of ZIP6 was made with a high degree of confidence being supported by the presence of 16 tandem MS spectra, with 11 high-energy collision-induced dissociation fragment spectra passing stringent filtering criteria for relative quantification of their TMT signature ion profiles. ZIP10 represented the second most abundant protein in the dataset, exhibiting enrichment ratios that indicated its reliance on ZIP6 for co-purification. In contrast, the protein Titin (aka connectin) was also confidently identified but co-isolated non-specifically with affinity matrices, as evidenced by TMT ratios near 1.0 (log_2_) in all TMT ratio calculations (Figures 7B and 8).

## DISCUSSION

In the present study we show that ZIP10 can stimulate cells to undergo EMT through inactivation of GSK-3 and down-regulation of CDH1. More importantly, we show that this occurs not only in a breast cancer cell line, but also in zebrafish embryos in which Zip10 stimulates Cdh1 down-regulation and cell autonomous migration responsible for gastrulation and anterior-posterior axis formation. All these ZIP10-related observations are very similar to data previously reported for ZIP6 [4,23,50]. Evidently, both Zip6 and Zip10 need to be present for proper EMT during zebrafish epiboly and the conclusion from this is that Zip6 and Zip10 must operate as a unit in this process. Indeed, we demonstrate that ZIP6 and ZIP10 exist as a heteromer and can likely function as a native complex. It has been indicated before by immunoblots that endogenous ZIP6, ZIP7 and ZIP10 can form homodimers [5,23,52] and recombinant ZIP5 and ZIP13 have been characterized to homodimerize [53,54], but to our knowledge this is the first study to show that two different protein members of the ZIP family of zinc transporters can operate as a heteromeric complex.

ZIP6 and ZIP10 being able to form a functional zinc-transporting unit explains a number of observations. The perhaps most compelling of these is that the morpholino-based knockdown of either *zip6* [4] or *zip10* in the zebrafish embryo inhibits EMT during the gastrulation process and results in very similar phenotypes, suggesting that they are not functionally redundant. To operate as a complex their expression is coordinated through Stat3 transactivation [4,6,23]. Knockdown of *zip10* in zebrafish embryos resulted in up-regulation of mRNA for both *stat3* and *zip6*. It may be speculated that this is a compensatory response to restore Zip6:Zip10 mediated EMT during zebrafish gastrulation. Recently published results give evidence for an even more diverse role for ZIP6 and ZIP10 as a functional moiety. The mouse oocyte rapidly accumulates zinc during the final phases of maturation [55]. Intriguingly, this zinc influx is mediated by maternally derived ZIP6 and ZIP10, and is required for progress through telophase I and subsequent arrest in metaphase II [55,56]. Thus, in a variety of cell types and physiological states ZIP6 and ZIP10 are co-expressed and participate in the same molecular and cellular processes.

ZIP6 and ZIP10 stimulate motility in cells at least in part by down-regulation of CDH1, through a signalling pathway which may involve the inactivation of GSK-3 through serine phosphorylation and transcriptional repression by SNAIL [4,23]. In the present study, forced expression of ZIP10 in MCF-7 cells resulted in increased abundance of the inactive serine phosphorylated GSK-3α and GSK-3β. According to the canonical pathway, active GSK-3β phosphorylates SNAIL resulting in export from the nucleus, ubiquitination by β-TRCP and degradation through the proteasome pathway [57]. However, GSK-3 also inhibits SNAIL transcription in human cells and it has been reported that inhibition of both GSK-3α and GSK-3β isoforms is required for increased SNAIL expression [58]. Interestingly, we have recently discovered that both GSK-3α and GSK-3β co-immunoprecipitate with the ZIP6:ZIP10 complex (PMD: 12083774), consistent with the interpretation that their biology might be integrated and might involve the zinc-dependent regulation of these kinases.

Zip10 is required for zebrafish development because morpholino-based knockdown of its respective mRNA produced a severe developmental morphant phenotype typified by shorter anterior-posterior axis at 24 hpf. The embryo at 24 hpf was runted, generally under-developed, and was very similar to the *zip6* morphant zebrafish described before [4]. This observation is consistent with the reported expression of the *zip10* gene very early in life in the zebrafish embryo [59]. Central nervous system abnormalities, including a severely dysmorphic head and missing or small eyes, were observed in most affected embryos with shorter and thickened anterior-posterior hypoblast. These deformities were similar to the ones obtained by *zip6* or *stat3* knockdowns [4,60] and, clearly, Zip6 and Zip10 are both required for cell migration responsible for anterior-posterior axis formation. Some of the defects observed in the Zip10^MO^ zebrafish were also found following morpholino knockdown in zebrafish of Zip7 [61], which mediates gated zinc(II) flux from the ER [5] and the Golgi apparatus [62]. Specifically, knockdown of Zip7 also resulted in embryos with decreased size of the head and eye, and shorter and curved spinal cord [61], showing that several zinc transporters contribute to formation of these structures.

Although loss of Cdh1 is associated with EMT during zebrafish gastrulation, it has also been shown that Cdh1 is required for epiboly, as well as convergence and extension cell movements [63,64], where it connects deep cell and enveloping cell layers. During epiboly, a programme executed during zebrafish development between 4 and 10 hpf, a multi-layered sheet of ectodermal cells spreads over and encloses deeper cell layers, becoming thinner as a result [65]. It is interesting to note that the *zip10* morphants in the present study looked similar to Cdh1 mutants described previously (i.e. delayed epiboly, runted body and sometimes no tail) [65]. Considering the numerous signalling pathways involved in gastrulation, disrupting any of these may converge to a similar shortened anterior-posterior and broadened mediolateral body axes. Thus, these similar phenotypes could be the result of distinct mechanisms [66]. Our findings indicate a fundamental role of Zip10 in zebrafish embyogenesis, particularly in anterior-posterior body axis formation and in development of the heart and central nervous system. Because of the non-redundant nature of cellular functions carried out by Zip6 and Zip10 and their formation of a heteromer, it is possible that the observed up-regulation of *zip6* and *stat3* expression in Zip10^MO^ embryos was a compensatory response to counteract the loss of Zip10.

In addition to the phenotypic effects in common with those published for the *zip6* morphant [4], knockdown of *zip10* caused pericardial oedema and deformed string-shaped heart. Similar deformities in zebrafish were observed through manipulations of the zinc finger transcription factor, Tbx5 [67,68]. It was also shown in rats that zinc deficiency in the dam results in foetuses with heart anomalies because of the alterations in the expression and distribution of several proteins involved in its development, including human natural killer-1 (HNK-1), Connexin-43 (Cx43), survival of motor neuron 1 (SMA), GATA-4 and FOG-2 [69].

The evolutionary and general relevance of the findings in the present study are underlined when considering that, based on sequence homology, *Drosophila* has only a single orthologue representing the vertebrate *zip6* and *zip10* genes, known as *fear-of-intimacy* (FOI). Like its vertebrate orthologues, FOI is essential for proper cell migration during embryogenesis [70]. More specifically, FOI has been shown to be required for morphogenesis of gonads and trachea, and controls glial cell migration [71–73]. Thus, the role of ZIP6/ZIP10 in executing EMT is evolutionarily conserved from fly to man.

The effects of a particular zinc flux on cellular signalling can be the result of its movement of zinc(II) across cellular membranes or may reflect its ability to interact with other proteins. For instance, members of the ZIP6/10-containing sub-branch of ZIP zinc transporters have been shown to interact with the PrP (prion protein), and were subsequently shown to have a common ancestry with the prion gene family [74,75]. Consistent with their evolutionary relationship, members of this ZIP branch comprise at their N-terminus a PrP-like ectodomain that appears to contain elements required for dimerization, a feature that might also explain the ability of these proteins to interact with PrP [54]. It has been proposed that PrP plays a role in cell adhesion by regulating CDH1 delivery to the cell membrane, which in turn controls the formation of adherens junctions. Moreover, the knockdown of PrP in a zebrafish gastrulation model or its knockout in a mammalian model of EMT interferes with autonomous cell movements and the execution of EMT [76–78]. This observation is consistent with the independent findings that ZIP6 and ZIP10 are important for cell autonomous anterior mesendodermal cell movement. Thus, the present study adds to an emerging body of data, which increasingly supports the conclusion that ZIPs carrying PrP-like ectodomains and their PrP molecular cousin [78] are integral to and essential for cellular plasticity programmes, which are activated when cells are mobilized.

## Abbreviations

CDH1/E-cadherin: epithelial cadherin
CHO: Chinese-hamster ovary
EMT: epithelial–mesenchymal transition
ER: endoplasmic reticulum
GSK: glycogen synthase kinase
hpf: hours post fertilization
IRT1: iron(II) transport protein 1
ISH: *in situ* hybridization
Morpholino: morpholino-modified antisense oligonucleotide
PLA: proximity ligation assay
PM: plasma membrane
PrP: prion protein
qPCR: quantitative real-time polymerase chain reaction
STAT: signal transducer and activator of transcription
TMT: tandem mass tag
β-TRCP: β-transducin repeat containing E3 ubiquitin protein ligase
ZIP: Zrt- and Irt-like proteins
